# Preserved Modular Network Organization in the Sedated Rat Brain

**DOI:** 10.1371/journal.pone.0106156

**Published:** 2014-09-02

**Authors:** Dany V. D'Souza, Elisabeth Jonckers, Andreas Bruns, Basil Künnecke, Markus von Kienlin, Annemie Van der Linden, Thomas Mueggler, Marleen Verhoye

**Affiliations:** 1 F. Hoffmann-La Roche Pharmaceuticals Ltd, Neuroscience Discovery, Basel, Switzerland; 2 Bio-Imaging Lab, University of Antwerp, Antwerp, Belgium; University of Maryland, College Park, United States of America

## Abstract

Translation of resting-state functional connectivity (FC) magnetic resonance imaging (rs-fMRI) applications from human to rodents has experienced growing interest, and bears a great potential in pre-clinical imaging as it enables assessing non-invasively the topological organization of complex FC networks (FCNs) in rodent models under normal and various pathophysiological conditions. However, to date, little is known about the organizational architecture of FCNs in rodents in a mentally healthy state, although an understanding of the same is of paramount importance before investigating networks under compromised states. In this study, we characterized the properties of resting-state FCN in an extensive number of Sprague-Dawley rats (n = 40) under medetomidine sedation by evaluating its modular organization and centrality of brain regions and tested for reproducibility. Fully-connected large-scale complex networks of positively and negatively weighted connections were constructed based on Pearson partial correlation analysis between the time courses of 36 brain regions encompassing almost the entire brain. Applying recently proposed complex network analysis measures, we show that the rat FCN exhibits a modular architecture, comprising six modules with a high between subject reproducibility. In addition, we identified network hubs with strong connections to diverse brain regions. Overall our results obtained under a straight medetomidine protocol show for the first time that the community structure of the rat brain is preserved under pharmacologically induced sedation with a network modularity contrasting from the one reported for deep anesthesia but closely resembles the organization described for the rat in conscious state.

## Introduction

Resting-state functional magnetic resonance imaging (rs-fMRI) has gained widespread attention for investigating the intrinsic organizational structure of brain neurocircuitry by probing the dynamic relationship between brain regions which has been termed functional connectivity (FC; for review see van den Heuvel and Hulshoff Pol [1]). Unlike traditional task-based fMRI, rs-fMRI is based on the spontaneous fluctuations of the blood oxygenation level dependent (BOLD) signal at rest and their temporal correlation across anatomically separated brain regions [2], [3]. Functional connectivity networks (FCNs) sub-serving not only sensorimotor functions but also higher order cognitive capabilities have been identified in humans [3]–[7]. The technology is currently evaluated for its diagnostic and potentially prognostic value for elucidating the relationship between abnormal FC and the symptomatology present in neurological and neuropsychiatric disorders such as Alzheimer's disease [8]–[10] or schizophrenia [11], [12] as well as in autism spectrum disorders [13].

Recently, translation of rs-fMRI applications to small laboratory animals has been reported (for review see [14] offering the possibility to investigate the neurophysiological basis, address potential confounding effects of physiological noise on BOLD-based FC measures, or to study the dynamic of the identified networks [15]–[19]. Furthermore it enables to probe FC within disease-relevant brain networks as recently demonstrated in experimental rodent models of peripheral nerve injury [20], spinal cord injury [21], stroke [22], absence seizure [23] and potentially in genetic mouse models [24].

Provided that disease-specific alterations can be detected with sufficient sensitivity rs-fMRI will eventually allow normalization (or rescue) of such FC abnormalities to be tested during pharmacotherapy [25]. In these rodent studies several analytical strategies have been employed to probe FC between brain regions. Utilizing a model-dependent seed approach, region-specific FCs have been discovered for somatosensory, motor, visual, prefrontal and retrosplenial cortical regions, as well as for subcortical caudate putamen and thalamic regions [26]–[30].

More recently, using a multivariate statistical approach (independent component analysis, ICA), it was demonstrated that, similarly to what has been described in human rs-fMRI, many neuroanatomical systems in the rat and mouse tend to be highly coherent in their spontaneous activity [31]–[35]. Seed-based approaches test specific hypotheses in relation to a brain region of interest, whereas model-free ICA approach divides the BOLD signal into different independent sources, or components revealing spatiotemporal patterns in the data. However, these approaches are both not optimal for studying complex organizational architecture of the brain.

An interesting and upcoming approach, which enables to derive information on the overall organization of the functional brain network, is the application of graph analysis to rs-fMRI time-course data [36]. In graph theoretical analysis, a complex FCN is treated as a graph of nodes and links, wherein the brain regions are represented by nodes, and the links (or edges) reflect the presence or degree of mutual correlation of their rs-fMRI responses. Various graph metrics (e.g. modular architecture, clustering coefficient or small-worldness) have been proposed to characterize both local and global properties of the brain FCNs [37], [38] and there is first evidence that such measures are altered in neuropsychiatric disorders e.g. in Schizophrenia [39], [40].

Rs-fMRI studies based on graph theoretical approach have so far prevalently focused on examining the FCNs in humans. However, only recently, Liang and colleagues [34] constructed rat brain FCNs from ICA components; they applied graph theory to examine the interaction between these components, and subsequently derived three community structures linking them to sensorimotor, autonomic regulation, and cognitive processing, respectively. However, like many other graph-based studies in humans [41]–[43], binary FCNs were constructed by retaining only the strongest positive connections. Consequently, this approach results in a loss of connectivity information. Furthermore, negative functional connections (anti-correlations [44]) between the independent components were not taken into account. Recent evidence suggests that also the negative FC between brain regions bears neurophysiological relevance [45]–[47].

In this study, we acquired rs-fMRI data in Sprague Dawley rats and performed partial correlation analysis based on the time courses between 36 brain regions. We constructed for the first time fully connected, positively and negatively weighted FCNs of the rat brain and applied graph measures proposed by Rubinov et al. [48] which overcome the limitations associated with thresholding (binarizing) and dealing with negative FCs (or anti-correlations).

Moreover by acquiring data in an extensive number of subjects (n = 40) under fully standardized conditions we were able to test for inter-subject reproducibility both on the FC outcome as well as at the level of the graph analysis. Aiming furthermore to deepen our understanding of the brain network organization specifically under sedation and to contrast it from the awake as well as anesthetized state we generated all data under a straight medetomidine protocol without concomitant administration of isoflurane to avoid reaching a level of deeper and general anesthesia.

## Material and Methods

### 1 Ethics Statement

All animal procedures were conducted in strict adherence to the Swiss federal regulations on animal protection, according to the rules of the Association for Assessment and Accreditation of Laboratory Animal Care International (AAALAC), and with the explicit approval of the local veterinary authority.

### 2 Animal preparation and physiological monitoring

40 male Sprague-Dawley rats (Charles River, France) weighing 250–270 g, were group housed and maintained in temperature-controlled conditions with a 12 h light/dark cycle (6 a.m.–6 p.m., lights on). Food and water were provided *ad libitum*.

Prior to MRI experiments animals were weighed and anesthesia was initiated with 4% isoflurane (Abott, Switzerland) in a mixture of oxygen (0.2 l/min) and air (1.0 l/min) administered in a inhalation chamber. Following a subcutaneous bolus injection of medetomidine at a dose of 0.2 mg/kg (Dorbene, Graeub AG, Switzerland) rats were positioned – under a continued 2% isoflurane anesthesia - in the MR scanner in a cradle made of fiberglass with the head immobilized in a stereotaxic holder with tooth and ear bars. Body temperature was measured rectally and maintained at 37°C using a feedback-regulated electric heating blanket. Breathing rate and concentrations of inhaled and exhaled oxygen and CO_2_ were continuously monitored on a PowerLab data acquisition system (ADInstruments, Germany). After positioning, the delivery of isoflurane was stopped and a continuous subcutaneous (s.c.) infusion of medetomidine (0.1 mg/kg/h) was started. Total exposure time to isoflurane did not exceed 8 minutes and was discontinued 1 hour before first resting-state data acquisition. At the end of the MR assessment medetomidine was antagonized by a single s.c. injection of atipamezole (0.2 mg/kg) (Alzane, Graeub AG, Switzerland).

### 3 MRI acquisition

The experiments were performed on a 9.4T/20 cm Biospec scanner (Bruker BioSpin, Germany) equipped with transmit volume coil and a quadrature receive-only surface coil tailored for rat head.

First, a set of scout images (*T*_2_-weighted rapid acquisition relaxation-enhanced (RARE) sequences [49]: repetition time (TR)  = 1.8 s, effective echo time TE*_eff_*  = 35.94 ms, RARE-factor 8, 256×128 matrix, 2 averages) in three orthogonal planes were acquired in each animal, in order to locate the most rostral extension of the corpus callosum, which served as landmark for positioning image planes in the subsequent scans. Anatomical images were obtained (*T*_2_-weighted: RARE TR/TE*_eff_*  = 2.5 s/33.44 ms, RARE-factor 8, field of view (FOV)  = (3.2×3.2) cm^2^, 256×256 matrix) from 20 coronal slices (slice thickness  = 1 mm) used for co-registration of functional images.

Resting-state data were acquired using a *T*_2_*-weighted single shot gradient echo planar imaging (GRE-EPI) sequence with TR/TE 2 s/17.5 ms. Twenty coronal slices of 1 mm thickness, an interslice distance of 0.1 mm, FOV of (3.2×3.2) cm^2^ and matrix size of 128×128 were acquired with the same slice position as the anatomical RARE images. 165 EPI volumes were acquired per subject leading to a duration of 5.5 min per run.

### 4 Pre-processing of MRI data

**Figure 1:**
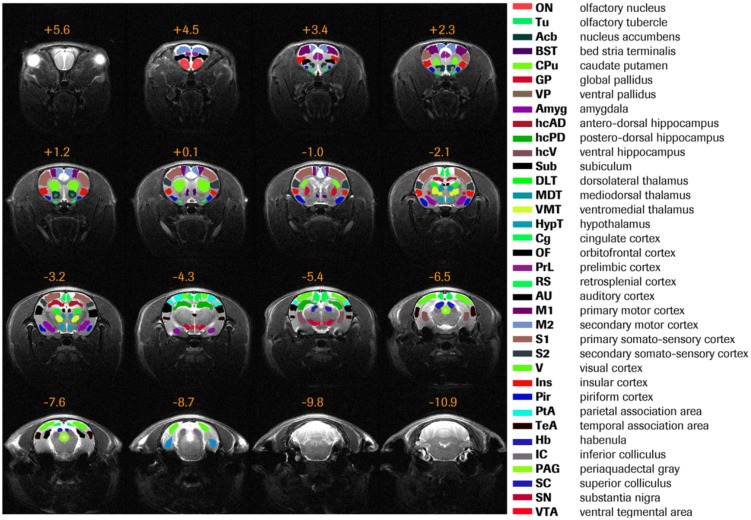
Rat brain parcellation scheme. Thirty six anatomically defined brain regions of interest, colored differently, are overlaid on the in-house rat MRI template. Distance to Bregma (in mm) is given at the top of each slice.

All MRI data were pre-processed using FSL (FMRIB Software Library; http://www.fmrib.ox.ac.uk/fsl). First, both anatomical and functional datasets underwent brain extraction employing FSL's BET function. The following steps were used to preprocess the functional EPI datasets: motion correction, high-pass filtering (>0.007 Hz), regression of motion parameters. EPI datasets were registered to an in-house rat brain MRI template prior to in plane spatial smoothing (FWHM 0.5×0.5 mm^2^). The applied parcellation scheme and list of region-of-interest (ROI) is provided in Figure 1. ROIs were defined on the in-house template making use of an in-house atlas which was manually delineated based on the Paxinos atlas [50].

### 5 Data Analysis and statistical measures

#### 5.1 Construction of fully-connected Functional Connectivity Networks (FCN)

To estimate the direct functional connectivity (FC) between the 36 brain regions as defined by the rat brain atlas, and to construct FCNs from these connections we first extracted the mean rs-fMRI time course from each ROI in each subject by averaging over all voxels within individual regions using FSL's *fslmeants* function followed by partial correlation analysis of the rs-fMRI time courses between each pair of brain regions. This step resulted in a 36×36 matrix of partial correlation coefficients for each subject, where matrix elements represented the direct FC between pairs of brain regions. Correlation coefficients were converted into Fisher's z-values before applying algebraic and statistical operations. To assess between-group reproducibility of FC, the 40 subjects were randomly split into four groups (assuming that n = 10 represents a typical group size in standard study designs) and the FC matrices averaged within each group. The Pearson correlations between all pairs of groups across FC values of corresponding functional connections were calculated.

Next, FC matrices were averaged across all subjects to produce a final, mean FC matrix that comprised both positive and negative correlation coefficients indicative of positive and negative functional connections between brain areas. Here, the mean FC matrix represents a complex functional network of brain regions; in a mathematical sense this network can be conceptualized as a fully-connected weighted graph of brain regions serving as nodes, and edges represented by the weighted partial correlation coefficients. The fully connected, undirected rat FCN of 36 brain regions had 630 functional connections comprising both positive and negative weights.

#### 5.2 Graph properties

Elementary measures of the network graphs, computed each for positive and negative connections, as described by Rubinov et al. ^48^ are summarized as follows: Degree of a node *i* 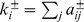, is the total number of positive or negative connections of node *i*; *a_ij_* denotes the weighted connection (partial correlation coefficient) between nodes *i* and *j*, and could be either positive or negative. Strength of node *i* 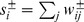 is the sum of positive or negative connection weights of node *i*; *w_ij_* denotes positive or negative weights associated with the connection between nodes *i* and *j*. Finally, total weight, 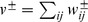 is the sum of all positive or negative connection weights of nodes *i, j* of the network. For all the graph analyses we used the MATLAB (Mathworks, Inc., Sherborn, MA) based functions of the Brain Connectivity Toolbox (https://sites.google.com/a/brain-connectivity-toolbox.net/bct/home) by Rubinov et al. [48].

#### 5.3 Identification of FCN modules

Modularity partition is defined as the complete segregation of the network into non-overlapping sub-networks or modules. To identify modular organization within the rat FCN we applied an algorithm recently proposed by Rubinov et al. [48] that aims at faithfully partitioning the FCN into modules by maximizing an objective function known as *modularity index* (Q*); here, Q* [0,1] quantifies the goodness with which a network can be partitioned into modules. For a fully-connected FCN with positive and negative weights the generalized modularity function is expressed as
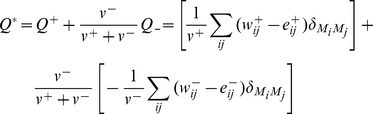



In the above equation the modularity measure *Q^+^*, which reflects the contribution of positive connection weights, is the average difference present between within-module connection weights 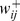 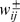 and chance expected within module connection weights 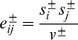, 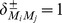 when *i* and *j* are in the same module and 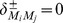 otherwise. The factor 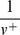 rescales the maximized 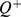 to the range of [0, 1]. Similarly, the analogous modularity measure 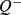 [0, 1], reflects the contribution of negative weights. Here, the definition of Q^*^ is based on the assumption that negative connections in the functional networks are important, albeit not equally important as positive connections, the term 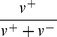 makes the contribution of negative weights auxiliary to the contribution of positive weights. According to Rubinov et al. [48] high Q* partitions should theoretically have most positive weights within modules, and most negative weights between modules.

To determine the robustness of the modularity structure of a FCN across subjects, the modularity affiliation vector (i.e. vector specifying module class for each network node) was computed for each individual subject. Next, we quantified the similarity between modularity affiliation vectors for each pair of subjects by means of their mutual information (MI) as well as the variation of information (VoI). To assess reproducibility at the group rather than at the individual level, mutual information as well as the variation of information were also determined between modularity affiliation-vectors obtained from group-mean FC matrices after randomly splitting the 40 subjects into 4 groups, as done before with the FC matrices themselves.

#### 5.4 Centrality of brain regions

Efficient routing of information within the brain network is governed by so-called central brain regions of strong functional connections to diverse brain regions. Such properties of brain regions in the FCN with positive and negative connections can be captured by two measures, namely *strength* and *diversity*. These measures were computed on the mean FC matrix. Here, the generalized strength of node *i* is given by 
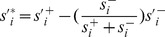
, where 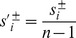 is the normalized connection strength; as before, the term 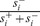 rescales the contribution of negative connection strength to meet the requirement that the positively weighted connections are more important than the negatively weighted connections. Similarly, the generalized diversity of node *i* is given by
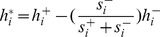
, where 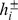 is the normalized connection diversity 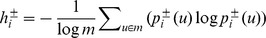, where 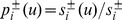, where 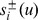 is the strength of node *i* within module *u*, and *m* is the number of modules in the modularity partition *M*; 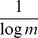 is a scaling factor). In this study, we classified brain regions as network hubs if their generalized strength and diversity were simultaneously greater than the respective mean value across all regions.

## Results

### 1. Functional Connectivity (FC) between brain regions

**Figure 2:**
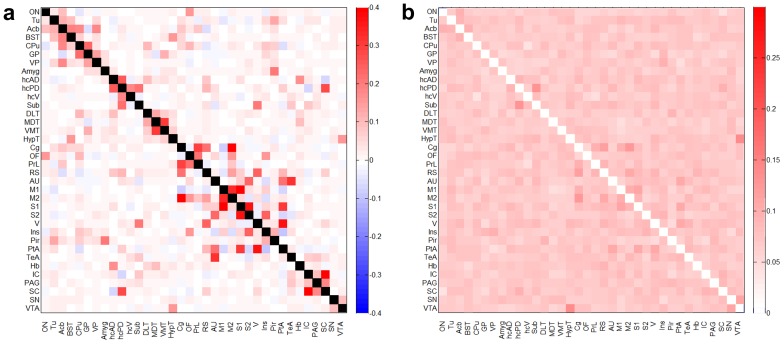
Fisher z-transformed FC matrix of rat brain. a) Mean FC matrix (40 subjects) showing pairwise partial correlation coefficients obtained for 36 brain regions. b) Standard deviation matrix of the partial correlation coefficients. For abbreviations of brain regions see Figure 1.

Partial correlations between the rs-fMRI responses across all pairs of brain regions were computed to assess the direct FC between brain regions. Matrices in Figure 2a & 2b represent the mean values and standard deviations of z-transformed FC computed between brain region pairs. Both positive and negative mean FC values were observed implying that the brain regions are functionally correlated as well as anti-correlated at rest, albeit the number of anti-correlations in the network was lower.

### 2. Robustness of FC pattern across groups of subjects

**Figure 3:**
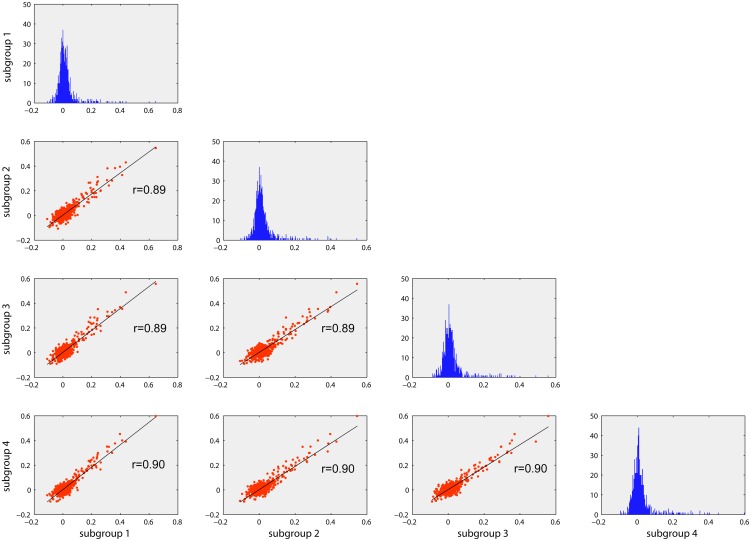
Reproducibility of FC across subjects. The histograms (in blue) represent the distribution of the partial correlation coefficients (z-transformed) for each of the four randomly partitioned subject groups. Scatter plots indicating high correlation (r>0.85) of the pair-wise FC values between the groups suggest high reproducibility of FC at a group level.

The plots, as represented in a half matrix format, in Figure 3, summarize reproducibility of FCs across subject groups. To this end, the 40 individual FC matrices were randomly split into four groups and averaged within each group. Plots on the diagonal of the matrix represent histograms of the FCs, which are similar across four groups. The scatter plots in the lower triangular part of the matrix represent correlations among inter-regional FC values, for all pairs of groups. High correlation values (r>0.80; p<0.001) indicate a good reproducibility of the direct FC pattern between brain regions.

### 3. Modular organization of rat FCN

**Figure 4:**
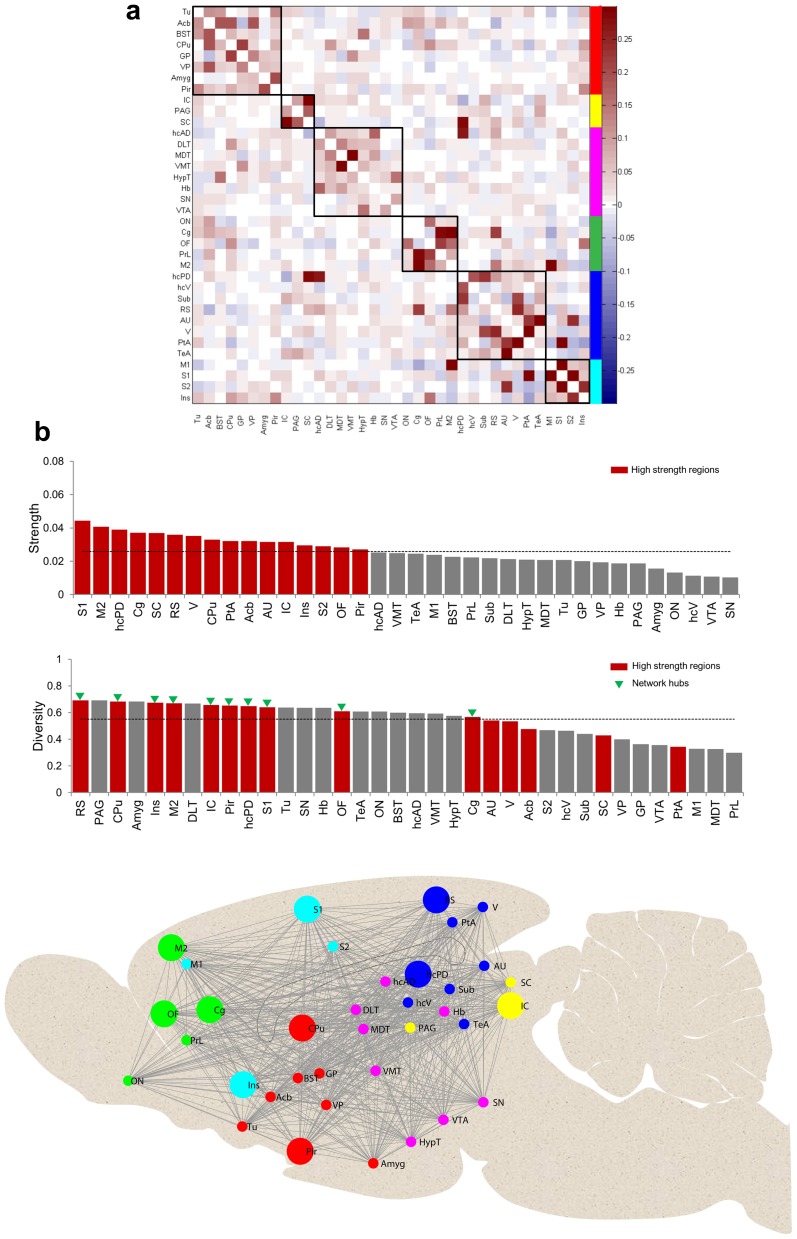
Modular organization of rat FCN and centrality of brain regions. a) Mean FC matrix rearranged with functional modules labeled along the major diagonal of the matrix. Six functional modules (sub-networks) of brain regions, as identified by the modularity partitioning algorithm (see methods), are labeled in different colors. b) In the first row brain regions are shown in descending order of their generalized connection strength. Strong (strength>mean) brain areas are shown in red bars; the horizontal line indicates the mean strength. In the second row brain regions are shown in descending order of their generalized diversity value. Here, red bars show brain regions with strong connections as determined in the first row. Network hubs (i.e., nodes that are both strong and diverse) are indicated by green triangles. c) FCN modules are visualized on a schematic mid sagittal section of the rat brain. Network hubs are depicted by larger circles. For abbreviations of brain regions see Figure 1. The colors used for the different modules are the same as those used in Figure 4a.

To detect modular organization in resting-state FCN we applied the modularity algorithm proposed by Rubinov et al. ^48^ on the mean fully-connected FC matrix with both positive and negative weights. The modularity function partitioned the FCN into six modules by achieving a maximum modularity index of Q* = 0.39. Figure 4a shows the six observed unequally sized modules of brain regions with strong functional connections, mostly positive in nature, whereas the inter-modular functional connections are rather weak or negative. Among the six modules, two modules comprised entirely cortical structures. A frontal module, labeled green, included the olfactory nucleus (ON), cingulate (Cg), orbitofrontal (OF), prelimbic (PrL), and secondary motor (M2) cortices. The second module, labeled cyan, included somatosensory (S1, S2) insular (Ins), and primary motor cortex (M1). A third module, marked in blue encompassed caudal structures of the cortex such as retrosplenial (RS), auditory (AU) and visual (V) cortex and association areas (parietal PtA, temporal TeA) as well as the hippocampal complex including posterior dorsal hippocampus (hcPD), ventral hippocampus (hcV) and the subiculum (Sub). Similarly, the fourth module labeled red included both cortical (piriform (Pir), olfactory tubercle (Tu), and subcortical structures (caudate putamen (CPu), nucleus accumbens (Acb), bed nucleus of stria terminalis (BST), globus pallidus (GP), ventral pallidus (VP), and the amygdala (Amyg)). The two remaining modules extended completely across subcortical structures. The smallest module, labeled yellow, comprised inferior and superior colliculus (IC and SC), and periaqueductal gray (PAG). The subcortical module colored in magenta comprised anterior dorsal hippocampus (hcAD), dorsolateral (DLT), mediodorsal (MDT) and ventro-medial (VMT) parts of the thalamus, as well as hypothalamus (HypT), habenula (Hb), substantia nigra (SN), and ventral tegmental area (VTA).

### 4. Centrality of brain regions

We computed two measures of centrality (strength and diversity) to characterize the role of individual brain regions within the rat FCN. Regions with connection strength above the mean (as obtained by averaging strengths across all brain regions) were classified as *high strength regions*. To further distinguish the role of individual nodes in terms of their intra- and inter-modular connectivity, we measured their diversity coefficients. In Figure 4b brain regions are shown in descending order of their generalized connection strength and diversity. It can be observed that not all brain regions which have high functional connection strengths also show high functional connection diversity. In this study, brain regions having concomitantly above-average strength and above-average diversity were classified as *network hubs* (cortical areas: somatosensory (S1), motor (M2), cingulate (Cg), orbito-frontal (OF), insular (Ins), piriform (Pir), retrosplenial (RS); subcortical areas: caudate putamen (CPu), posterior dorsal hippocampus (hcPD), inferior colliculus (IC)). Figure 4c provides a schematic overview of the overall topological organization of modules and hubs in the rat FCN.

### 5. Reproducibility of modular organization across subjects

**Figure 5:**
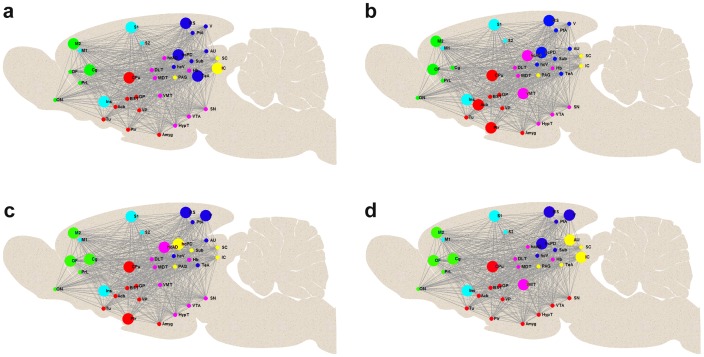
Modular organization in 4 different groups. Group plots with high modular similarity (low variability) in subjects randomly split into four groups (a–d) visualized on schematic mid sagittal sections of the rat brain. Network hubs are depicted by larger circles. The colors used for the sub-networks of the six functional modules, as identified by the modularity partitioning algorithm (see methods), are analogue to those used in Figure 4c. For abbreviations of brain regions see Figure 1.

To determine the robustness of the FCN modularity structure across individual subjects, we quantified the similarity of modular organization among subjects by determining MI and VoI for all pairs of subject modularity affiliation vectors. The mean MI and its standard deviation turned out to be 0.64±0.13, the VoI 0.34±0.12. By contrast, the corresponding values determined for 40 random networks (null networks based on each subject's FC matrix) were 0.15±0.03 and 0.68±0.04, respectively, confirming that the empirically observed MI and VoI values were substantially above and below chance, respectively. In order to perform an analogous assessment of modular variability at a group level, subjects were randomly split into four groups and FC matrices averaged within each group. Modular organization of the FCN in one example realization of such grouping is shown in Figure 5 a–d, respectively. In this case, modular organization in two groups (Figure 5 a&b) was identical and matched that one estimated from all 40 subjects, whereas two of the groups (Figure 5 c&d) showed a slightly different modular organization. To obtain a more general picture we tested potential confounding effect of sampling into subgroups and ran 1'000 such grouping randomizations, and determined MI and VoI values between pairs of group-level modularity affiliation vectors. The MI was 0.89±0.06 (standard deviation across group pairs and randomizations), the VoI 0.10±0.06 (null networks: 0.23±0.06 and 0.72±0.05), illustrating the effect of grouping at the FC-matrix level on reproducibility at the graph-metric level.

## Discussion

Reporting on the topological architecture of a fully connected resting-state FCN in rats, this study extends previous work on preclinical resting-state fMRI in which the analysis was based on ICA [31]–[35], seed-based correlation analysis approaches [26]–[30], or binary networks based on only positive weights ignoring the putative functional relevance of weaker and negatively weighted connections as well as anti-correlations [34], [47].

Resting-state fMRI data were acquired in spontaneously breathing, medetomidine sedated Sprague-Dawley rats and fully-connected large-scale complex networks of positively and negatively weighted connections were constructed based on Pearson partial correlation coefficients between time courses of 36 regions of interest encompassing almost the entire rat brain. Applying recently proposed complex network analysis measures Rubinov et al. [48], we show, that under the physiological condition of sedation the rat FCN exhibits a modular architecture, comprising six robust modules. In addition, we identified network hubs with strong connections to diverse brain regions.

As mentioned earlier, the FCN was constructed comprising also negative correlations. One possible explanation for the existence of these anti-correlations could be an inhibitory regulation of one region by another. Liang and colleagues [47] reported earlier on the existence of anti-correlations in the rat brain but focused only on a single connection (infralimbic cortex to amygdala), well-known to be anatomically densely interconnected and functionally inhibiting. In this study we extended this approach by exploring the overall brain network taking into account these negative correlations.

### 1. Characteristics of functional modules under sedation

Modularity and centrality measures as applied to the resulting FCN exhibit prominent community structure (or modules) as well as central nodes (Figure 4). A maximum modularity index of 0.39 was computed similar to previously reported results in a graph study based on binary networks [51]. Among the six identified modules in our study, four modules are characterized by containing exclusively cortical (2 modules) or subcortical (2 modules) regions, whereas the remaining two modules comprise regions from cortex and sub-cortex. Interestingly, a similar segregation into cortical and subcortical network clusters has also been proposed by Schwarz et al. [52] based on connectivity measures using pharmacological MRI and by Liang and colleagues [34] who investigated the intrinsic modular architecture of the rat brain on the basis of rs-fMRI data.

Comparing the topological organization of brain networks identified in rats subjected to different states of consciousness Liang et al. [51] reported six modules under awake conditions and a reduced number of four modules under isoflurane induced general anesthesia. They furthermore observed a tendency towards enhanced segregation into exclusively cortical or subcortical modules under isoflurane when compared to the awake condition suggesting compromised communication between cortex and sub-cortex during anesthesia and accompanied loss of consciousness. Our data acquired under medetomidine with the identification of a robust modular organization show that the community structure of the rodent brain is conserved also under pharmacologically induced sedation. We identified the same number of modules (6) as reported for the awake state and the segregation into cortical and subcortical modules under medetomidine tended to be less exclusive than what was observed under isoflurane [51]. Medetomidine, a adrenergic α2 agonist, produces dose-dependent sedation and analgesia but leads only at high doses to a loss of consciousness, most likely via locus coeruleus noradrenergic neurons converging on the endogenous sleep-pathway [53]. It remains to be elucidated what level of unconsciousness is induced at the dose used in our study but based on our imaging findings it is tempting to speculate that animals subjected to the applied straight medetomidine protocol were in a condition of moderate or conscious sedation. To avoid any carry over effect of isoflurane onto the data acquisition period under medetomidine we kept firstly exposure to isoflurane at an absolute minimum required solely for induction purposes and secondly acquired the resting-state scans not before 60 minutes after discontinuation of the volatile anesthetic. Kalthoff and colleagues [54] recently showed that the occurrence of FC networks is heavily compromised under isoflurane at a level of 1.5% stating that this most likely results from global signal fluctuations caused by the isoflurane-induced burst-suppression-like neural activity. The lower number of modules and the near exclusive segregation into cortical and subcortical modules observed under isoflurane anesthesia [51] may therefore be related to a specific effect of isoflurane rather than representing a common feature of unconsciousness. Clearly, the neuronal mechanism underlying sedative- and anesthetic-induced loss of consciousness is different and each on itself is likely to be rather complex. As outlined nicely by Nallasamy and Tsao [55], FC mapping is highly appropriate to investigate the effects of anesthesia and sedation on the topological organization of the brain and studies using graph measures under different anesthetics and dose ranges are highly warranted, thus extending previous work on anesthesia-induced FC changes in which only seed-based analysis approaches have been used [56], [57].

As mentioned above also the measure of centrality applied to our FCN data exhibited a prominent community structure with occurrence of network hubs. Analysis of human brain connectivity has consistently identified a set of regions critically important for enabling efficient neuronal signaling and communication [58]. The central embedding of these candidate ‘brain hubs’ in anatomical networks supports their diverse functional roles across a broad range of cognitive tasks and widespread dynamic coupling within and across functional networks. Importantly, this high level of centrality also renders these brain nodes points of vulnerability that are susceptible to disconnection and dysfunction in brain disorders. In rodent brain network analysis of FC data the feature of centrality has not been explored. In this study, we defined a region as network hub if it features a high centrality meaning that both, generalized strength and diversity of this region were greater than the respective mean value across all regions. Several cortical and subcortical network hubs have been identified and will be discussed in more detail in the section below along with the modular organization taking into account the reproducibility and variability of the modular organization.

### 2. Reproducibility of FC and modular organization

High mutual information and low variation of information, between the modularity partitioning vectors (obtained for each subject) for all subject pairs suggest reproducibility of modular organization across subjects. Similar estimations performed on forty random networks (null or reference networks based each subject FC matrix) indicate the absence of robust modular organization (i.e. low mutual information and high variation of information) as observed in the experimental data. Moreover, by dividing the total number of animals (n = 40) into 4 equally sized groups and comparing the FC outcome of the 4 groups to the overall FC outcome we could further estimate the reproducibility of the FC and modular organization. It became apparent that the overall brain connectivity and general partitioning in modules is reproducible with small deviations in the modular organization across the four groups which have to be taken into account when interpreting the overall modular organization represented in Figure 4c.

Moreover it was shown earlier that between-subject variability in resting state data is higher when animals are measured under awake compared to anesthetized conditions [59], [60]. Although these results were based on a ROI-based analysis it might well be that similar difference in variability between the different conditions is present when applying a graph based approach. However, in the present study we only measured and estimated reproducibility under sedation and cannot put the finding in relation to variability under awake or fully anesthetized condition but extension of our graph-based approach to different physiological and disease conditions is pursued.

We have already highlighted the similarity in terms of the number of modules and its segregation into cortical and subcortical clusters between our data and previous reports about community structure. Furthermore it is evident that the regions assembled in the different modules and their topological organization in our study in rats under the physiological condition of sedation resembles to a high degree the community structure observed in the awake rat [51]. Modules comprising only cortical regions were divided in a frontal module (green; with olfactory nucleus, orbitofrontal, cingulate and prelimbic, as well as secondary motor cortices) and a more medial located module (labeled cyan) comprising insular, somatosensory (S1 and S2) and primary motor cortex. This frontal cortex organization including its hub structure was highly reproducible across the four groups. Both cortical modules (green and cyan) comprised several network hubs: Secondary motor-cortex, cingulate cortex, orbito-frontal cortex, somatosensory cortex and insular cortex. The majority of their functional connections were coupled to areas within modules of the cortical ribbon indicating a strong intercortical communication as suggested already in previous work [34].

Similar to what has been found by Liang and colleagues under awake conditions [51] the module (labeled blue) with the most posterior located cortical regions (retrosplenial cortex, auditory and visual cortex) and association (parietal, temporal) areas included structures of the hippocampal complex (posterior dorsal hippocampus, ventral hippocampus and subiculum). In contrary to their description of this module our analysis did not result in thalamic regions (dorso-lateral, medio-dorsal and ventro-medial thalamus) being part of this module but rather assembled in a sub-network together with hypothalamus, and midbrain structures VTA and SN. This - magenta labeled - module also comprised habenula and sub-regions of the hippocampus. Overall the boundaries between these two modules (labeled blue and magenta) - comprising mainly posterior cortical, hippocampal and midbrain subcortical regions - were less reproducible than the two frontal cortex modules and no clear hub structure became apparent. In one of the four groups (Figure 5d) the midbrain structures (VTA, SN and HypT) were part of a different module, the basal ganglia module (labeled red) comprising mainly striatal regions and amygdala (including stria terminalis). In this module the CPu was the most reproducible hub. The finding of CPu showing a high centrality may indeed reflect its central function within the cortico-striatal-thalamic loop integrating cortical, mesolimbic and nigro-striatal signals with emotional content and contextual information from amygdala and hippocampus, respectively. The smallest module (indicated in yellow) comprising only inferior and superior colliculus as well as PAG showed a good reproducibility with only one group (Figure 5b) and spread across other regions for the different groups.

Overall the data of the modular organization clearly show that the segregation into cortical and sub cortical modules is abrogated mainly by three structures. The piriform and olfactory cortex are sharing the same module and are strongly connected with structures of the basal ganglia. On the other hand albeit the modular affiliation of the hippocampus sub-regions showed the highest variability (sub-regions belonging to different networks) it became obvious that posterior dorsal parts of the hippocampus are strongly inter-connected with the posterior cortical areas. Interestingly, the observed frontal and posterior cortical modules (color coded blue and green, respectively) match to a very large degree the brain regions recently identified in a rs-fMRI study using ICA and proposed as the rat default mode network (DMN) [35] homologous to the one reported in humans [61]. In their work this network separated into two modules which remarkably closely resemble the frontal and posterior cortical (including the posterior hippocampus) modules (labeled green and blue, respectively) we are describing, except that in our data the auditory (AU)/temporal association cortex (TeA) are associated with the frontal module. Obviously such a direct comparison between the findings of the two studies is limited by the different anatomical parcellation chosen.

In general the information about reproducibility and variability of the modular organization represents not only valid information about what to expect investigating the brain's topological organization in normal rats. This inherent variability has to be taken into account when extending this approach to study potentially altered community structure or modularity under certain pathological conditions.

## Conclusions

To the best of our knowledge, this is the first functional MRI study in the rat to apply fully-connected complex network analysis approach aiming to explore the modular organization and hubness of the brain under resting conditions. We provide evidence that the overall rat FCN community structure is preserved under pharmacological induced sedation and highly reproducible between animals. These empirical findings will be important for conducting studies that aim at discerning the topological organization of resting-state networks in various models of central nervous system disorders.
